# Mix Optimization of Polymer-Modified Cementitious Composites: Combined Effects of FA-PFDS, Cellulose Nanofibers and Synthetic Fibers on Mechanical Properties and Water Absorption

**DOI:** 10.3390/ma19214473

**Published:** 2026-10-23

**Authors:** Jianzun Lu, Shengrui Wang

**Affiliations:** 1 Center for Coastal Zone Innovation and Development Research, Institute for Advanced Study, Beijing Normal University, Zhuhai 519087, China; 91132026002@bnu.edu.cn; 2 National Engineering Research Center of Advanced Technology and Equipment for Water Environment Pollution Monitoring, Beijing Normal University, Zhuhai 519087, China; 3 Guangdong-Hong Kong Joint Laboratory for Water Security, Beijing Normal University, Zhuhai 519087, China; 4 Guangdong Provincial Observation and Research Station for Water Resources and Ecological Environment in Dongjiang River Basin, Beijing Normal University, Zhuhai 519087, China; 5 College of Resources and Environment, Yunnan Agricultural University, Kunming 650201, China

**Keywords:** hydro-mechanical slope model, LASSO regression, microstructural mechanism, polymer-modified cementitious composite

## Abstract

Partially replacing cement with superhydrophobic fly ash (FA-PFDS), cellulose nanofibers (CNF) and synthetic fibers (SYN) is a promising route to combine mechanical performance and water resistance in polymer-modified cementitious composites. However, current studies rarely quantify the dosage sensitivity under the complex interactions of multiple polymers, which often results in trade-offs between mechanical strength and impermeability. This study employs an *L*_36_(6^3^) orthogonal experimental design combined with least absolute shrinkage and selection operator (LASSO) regression to analyze macroscopic performance data, supported by X-ray diffraction (XRD), scanning electron microscopy (SEM) and a simplified hydro-mechanical slope model to discuss possible mechanisms and structural responses. The LASSO models predicted that a mixture of 20% FA-PFDS, 1% SYN and 0.4% CNF by mass of cement replaced would reach a 7-day compressive strength of 18.20 MPa with a water absorption of 8.15% and a consistency of 16.3 mm while reducing cement consumption by 21.4%. XRD and SEM observations are consistent with capillary pore filling by CNF and crack bridging by SYN, but the proposed mechanism remains to be verified. The slope model suggests that a modified surface layer with reduced permeability would delay rainfall breakthrough of a thin surface layer and keep stresses well below the assumed 20 MPa load. Overall, this study provides empirical evidence on how the dosage of multiple polymers affects the trade-off of strength and impermeability, and a data-driven starting point for mix design in slope-protection applications, pending experimental validation.

## 1. Introduction

Concrete is the most widely used construction material worldwide, with annual demand approaching 30 billion tons [1,2]. Its main binder, ordinary Portland cement (OPC), is resource intensive and contributes substantially to environmental impacts. Cement manufacture consumes 12–15% of industrial energy and accounts for approximately 8% of global anthropogenic carbon dioxide (CO_2_) emissions [2,3]. The transition towards low-carbon binders is therefore an important priority for civil and structural engineering, particularly in the context of carbon neutrality and net-zero emission targets by 2050 [2,3]. Upcycling industrial and municipal solid wastes into polymers for partially replacing OPC in cementitious composites is one promising pathway [4]. This strategy can reduce landfill burdens and decrease cement consumption while supporting more sustainable engineering applications.

For engineering applications such as surface stabilization, pavement layers and slope protection, cementitious composites are required to resist both mechanical loading and water ingress. Their intrinsic porosity and hydrophilicity facilitate the ingress of water and harmful species, which can accelerate deterioration under wetting, ionic attack and cyclic weathering [5,6]. Solid waste-derived polymers, including geopolymer, biopolymer and synthetic polymer, have been used to improve the strength and water resistance of cementitious composites [4]. However, each polymer has limitations. For instance, geopolymers can reduce carbon footprint, but changes in porosity and pore connectivity may increase water absorption and compromise durability [4,7]. Synthetic fibers (SYNs) are chemically inert and hydrophobic, but weak bonding with hydration products can lower interfacial strength [4,8]. Biopolymers, such as cellulose nanofibers (CNFs), can fill pores, support internal curing and bridge microcracks, but their hydrophilicity can reduce the effective water-to-binder ratio. Excess CNFs may also cause water absorption, agglomeration or gelation [9]. These trade-offs show that the key challenge is not whether polymers can modify cementitious composites, but how different polymer types can be combined to overcome their individual limitations.

It is challenging to optimize combinations of multiple polymers in cementitious composites, because optimization means selecting FA-PFDS, CNF and SYN contents that reduce OPC consumption while maintaining unconfined compressive strength (UCS), limiting water absorption and preserving workable consistency. In this study, a combined effect is defined statistically as a non-additive interaction term between polymer contents in the fitted model. FA-PFDS reduces water uptake and contributes mineral phases, CNF fills capillary voids, and SYN improves interfacial crack bridging. Such interactions cannot be inferred from single-factor tests alone because polymer contents affect strength, consistency and impermeability through coupled main and interaction effects.

An interpretable optimization method is therefore required. Conventional mix design often relies on trial-and-error testing, which is inefficient for identifying synergistic and antagonistic effects among multi-polymers [10]. Machine learning (ML) has increasingly been used to predict the properties of cementitious composite [11,12], but many models have limited interpretability and are sensitive to multicollinearity in multidimensional datasets [13]. Least Absolute Shrinkage and Selection Operator (LASSO) regression is useful in this context because its *L*_1_ penalty can reduce overfitting, address multicollinearity and select influential variables. It is therefore suitable for quantifying the relative contributions of polymer contents and interaction terms in cementitious composites [10,13].

However, ML models cannot reveal the microstructural mechanisms or structural performance of cementitious composites [12]. A hybrid approach is therefore needed to link material-level optimization with structural performance. Interpretable regression can identify proportions of multi-polymer mixes in cementitious composites, microstructural characterization can explain the underlying mechanisms, and finite element modelling (FEM) can translate material properties into structural responses [12]. In particular, Abaqus/CAE can incorporate density, elastic modulus, porosity and permeability into structural models. Coupled hydro-mechanical elements can simulate rainfall infiltration, whereas three-dimensional solid elements can evaluate stress distribution and settlement under static loading. Combining these methods provides a route to discuss both the possible mechanisms and the engineering potential of polymer-modified cementitious composites.

This study aims to investigate the combined effects of multiple polymers, including FA-PFDS, CNF and SYN, on the microstructural and physicochemical properties of cementitious composite, thereby enhancing the mechanical and impermeable performances while achieving cement reduction and promising acceptable engineering quality. A hybrid approach is established that integrates orthogonal experimental design, LASSO regression, X-ray diffraction (XRD), scanning electron microscopy (SEM) and FEM. The objective was to identify a moderate combination of multiple polymers that reduces cement consumption while maintaining acceptable mechanical strength and moisture resistance of cementitious composite. This study further evaluates whether the polymer-modified cementitious composite can provide structural stability under rainfall infiltration and static loading to develop its potential of engineering application.

## 2. Materials and Methods

### 2.1. Materials

Ordinary Portland cement CEM I 42.5 R (Yongfa Construction Material Co., Ltd., Zhuhai, Guangdong Province, China) and standard quartz sand (Xiamen ISO standard Sand Co., Ltd., Xiamen, Fujian Province, China) were utilized as the primary binder and fine aggregate, respectively, to prepare the cementitious composites. Three solid waste-derived materials were incorporated as partial cement replacements, including FA-PFDS, CNF, and SYN, which were prepared in previous studies [7,8]. The FA-PFDS was fabricated through the surface functionalization of raw industrial fly ash (Henan Hengyuan New Material Co., Ltd., Zhengzhou, Henan Province, China) using 1H,1H,2H,2H-perfluorodecyltriethoxysilane (Shanghai Aladdin Biochemical Technology Co., Ltd., Shanghai, China). Meanwhile, CNF and SYN are waste-derived fibers obtained from municipal textile waste. CNF is the cellulosic fraction (diameter: 5–50 nm), which is reduced to nanofibrils by chemical oxidation, and SYN is the synthetic (polyester, diameter: 0.3–1 mm) fraction.

### 2.2. Orthogonal Design and Preparation of Polymer-Modified Cementitious Composites

The *L*_36_(6^3^) orthogonal design was used to investigate factorial effects on the responses, in which three factors were the replacement levels of FA-PFDS, CNF and SYN. All replacement levels are expressed as a percentage of the dry mass of cement in the control mixture. The level of FA-PFDS were 0, 10%, 20%, 30%, 40%, and 50%. The levels of CNF were 0, 0.4%, 0.8%, 1.2%, 1.6%, and 2.0%. For SYN, the levels were 0, 1%, 2%, 3%, 4%, and 5%. A total of 36 mixtures were prepared (Table 1), each tested in triplicate to ensure reproducibility and accuracy. The three modifiers replace cement mass for mass, and this cementitious composite is treated as the binder. The water-to-binder ratio is therefore 0.50 and the sand-to-binder ratio 3.0 in all 36 mixtures, so the water content is not a variable of the design. The components were mixed in a pot and stirred until thoroughly blended. The resulting mortar was placed into a 70.7 mm × 70.7 mm × 70.7 mm mold, vibrated 60 times and crisscrossed with a knife. Excess mortar was scraped off the top of the mold with a metal ruler, and the surface was smoothed horizontally three times. The mortar around the specimens was then wiped clean. The specimen age is counted as elapsed time from the first contact of the binder with water (t = 0). The filled molds were covered and kept at 20 ± 2 °C and 98% relative humidity for 24 h. The specimens were then demolded, marked and cured at 20 ± 2 °C and at least 95% relative humidity until t = 5 d, when the determination of water absorption and mechanical strength started.

### 2.3. Determining Consistency of Cementitious Composites

The consistency was measured with a Vicat apparatus following the procedure of GB/T 1346-2011, adapted to mortar by using the standard plunger. The penetrability of cementitious composites is different with various water contents. The consistency of cementitious composites can be determined by the penetration depth of the standard plunger.

Before determining the consistency of the cementitious composites, the sliding rod of a Vicat apparatus was ensured to move freely. The test mold and glass base plate were cleaned, and then the test mold was placed on the glass base plate. The pointer of the test rod was adjusted to zero when it touched the glass base plate. After each cementitious composite was prepared by mixing various components according to Table 1, a certain amount of cementitious composite was taken to fill the test mold immediately. The excess mortar was tapped five times lightly using a straight-edged knife to eliminate any voids. The excess mortar from the top of the mold was removed using a straight-edged knife to make a smooth surface. Then, the mold and base plate were moved to the center of the Vicat apparatus under the test rod immediately. The test rod was adjusted until it touched the surface of the cementitious composites. The screw was tightened for 1 to 2 s and then released suddenly. The distance between the test rod and glass base plate was recorded when the rod stopped sinking or after 30 s. The test rod was cleaned promptly for the next test. The entire operation should be completed within 1.5 min after mixing the cementitious composites.

### 2.4. Determining Water Absorption and Mechanical Strength of Cementitious Composites

In order to avoid ambiguity, the specimen age and conditioning are described explicitly. The specimen age is counted as elapsed time from t = 0, so that the water absorption and the UCS both refer to an age of 7 days. The conditioning was as follows: (i) from t = 5 d, the specimens were dried in an oven at 105 ± 5 °C for 24 h; (ii) at t = 6 d they were removed, cooled in a desiccator for 30 min to room temperature, and the mass of each cooled specimen was recorded as m_1_; (iii) the specimens were then immersed horizontally in deionized water at 20 ± 2 °C so that all six faces were in contact with water; (iv) at t = 7 d they were removed, surface-wiped and weighed (m_2_), and the water absorption was calculated using Equation (1); and (v) the UCS was measured on the same specimens at t = 7 d, within 1 h after the weighing, so that both properties refer to the same age of 7 days. The UCS is therefore that of specimens that were oven-dried and then saturated, not of specimens cured only in a moist atmosphere. This test measures the water uptake of dried, partly hydrated mortar.

(1) $$\mathrm{Water}\;\mathrm{absorption}\;\left(\%\right)=\frac{\left(m_{2}-m_{1}\right)}{m_{1}}\times 100$$

Finally, their unconfined compression strength (UCS) was measured using a hydraulic compression testing machine. A single cementitious composite was placed on the lower plate of the compression testing machine, ensuring it was centered. The load was applied uniformly at a rate of 2400 ± 200 N/s, ensuring continuous and smooth loading. After the cementitious composites failed, the maximum load applied to the cementitious composites at failure was recorded as F (N). The touch cross-sectional area was recorded as A (mm^2^), and the UCS of cementitious composites was calculated using Equation (2).

(2) $$\mathrm{UCS}\;\left(\mathrm{MPa}\right)=\frac{F}{A}\;$$

The average value of the measurements from the triplicates was taken as the determined value, with results accurate to 0.1 MPa. If the difference between either the maximum or minimum value among the three measurements and the intermediate value exceeded 15%, the intermediate value was taken as the determined value. If both the maximum and minimum values were different from the intermediate value by more than 15%, the test results for that group were considered invalid. Broken specimens were dried in an oven at 105 °C for 24 h, crushed and ground into homogeneous particles for instrumental analyses.

### 2.5. Microstructural Characterization of Cementitious Composites

Crystalline-phase mineralogy of the samples, from around 100 mg of each sample, was determined using a Bruker D8 Advance X-ray diffractometer (XRD, Billerica, MA, USA) in Hong Kong Baptist University, Hong Kong, according to JB/T 9400. The test was carried out with Cu-Kα (1.54 Å) radiation (40 kV–40 mA), with a detection range of 3–70° (2θ) and a step size of 0.02°.

For the SEM test, the microstructure of the samples was examined using ZEISS EVO18 Scanning Electron Microscope (Oberkochen, Germany) in Hong Kong Baptist University, Hong Kong. Representative freshly fractured surfaces of the samples were selected after gold plating under vacuum conditions.

### 2.6. Macrostructural Finite Element Modeling (FEM) Setup

**Figure 1:**
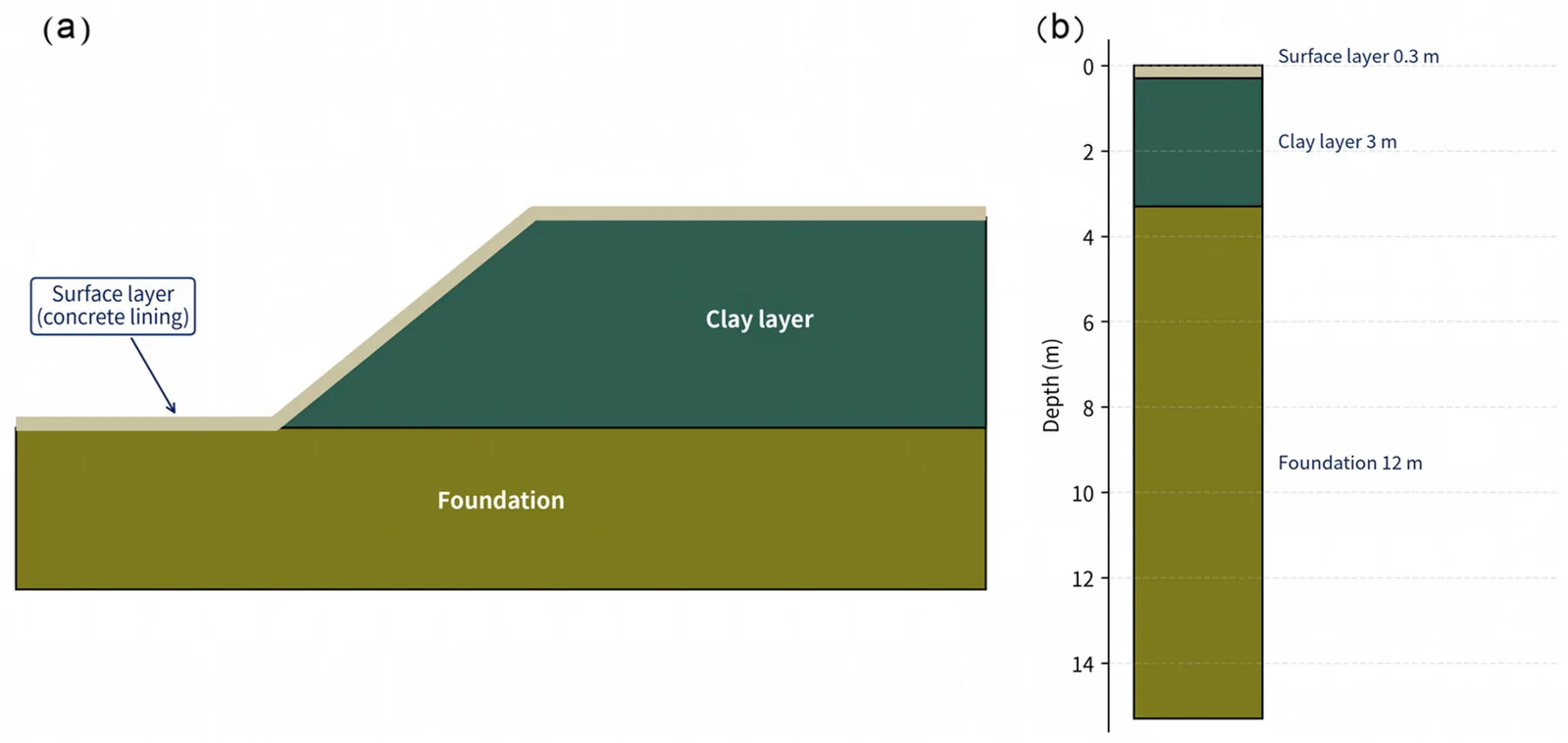
Three-layer slope model; (**a**) slop protection system including 0.3 m surface layer, 3 m clay layer, 12 m foundation; (**b**) Simulated column to scale.

The numerical model represents a slope protection system consisting of three layers: a 12 m foundation layer, a 3 m clay layer and a 0.3 m surface layer, as shown in Figure 1. The thicknesses follow common modelling practice: concrete lining and pavement surface layers are of decimeter thickness, and the clay layer covers the depth over which rainfall-induced pore-pressure changes are normally considered. The surface layer protects the clay layer from erosion. Two surface layers were compared: a conventional cement concrete layer and a modified layer in which 20% of the cement was replaced by FA-PFDS, 1% by SYN and 0.4% by CNF.

Material properties for each layer were taken from the literature and from previous experimental data of this study (Table 2). The properties of foundation and clay layer included typical base material characteristics such as the modulus of elasticity and Poisson’s ratio. The properties of surface layer included characteristics typical of soil stabilizers. Density, permeability and other relevant properties were included for each material, as shown in Table 2. These defined properties were then assigned to their respective sections of the model.

The model has two parts. (i) Rainfall infiltration: transient pore-pressure diffusion through the 15.3 m column was solved with a Crank–Nicolson finite-difference scheme under a ponded-water boundary condition at the top for 12 h, and the time at which the pore-pressure head at the base of the surface layer reached 50% and 90% of the applied head (breakthrough time) was recorded. The vertical displacement caused by infiltration was computed from the pore-pressure change with the one-dimensional constrained modulus M = E(1 − ν)/[(1 + ν)(1 − 2ν)]. (ii) Static loading: a uniform 20 MPa vertical stress was applied to the surface layer, and the lateral stress was obtained under zero lateral strain as σh = ν/(1 − ν) × σv, together with the compression of the 0.3 m surface layer. The 20 MPa load corresponds to the compressive strength level of the mortar and is used here as an upper-bound load case. The initial pore-pressure head was zero throughout the column; a constant head of 1 m was imposed at the top surface for 12 h and free drainage (zero head) at the base; interfaces between layers were assumed perfectly bonded with continuity of head and flux (harmonic-mean conductivity at the interface). All layers were treated as linear-elastic and no failure criterion was applied.

## 3. Results and Discussion

### 3.1. Combined Effects of Polymer Combinations on Mechanical Properties and Water Absorption of Cementitious Composites

The LASSO regression was used to describe the non-linear interactions among FA-PFDS, CNF and SYN. By adding an *L*_1_ regularization penalty, LASSO performs variable selection and shrinks coefficients, which helps limit overfitting in a small dataset (*n* = 36). The fitted coefficients quantify the contribution of each polymer content and of their interactions, and the fitted model was used to search for a mixture that gives a high strength with moderate water absorption and a reduced cement content.

In order to investigate the effects of three polymers on cementitious composite and to discuss the specific contribution of each polymer, the data obtained from orthogonal design (Table 3) were also analyzed using LASSO regression. According to the orthogonal design, cementitious composites were prepared, and their consistency was measured during preparation process. At an age of 7 days, the UCS and water absorption of cementitious composites were determined. The UCS of each specimen is a critical mechanical property, which indicates the quality and engineering application of the cement specimens. The water absorption affects the durability of cementitious composites. For instance, low water absorption suggests that cementitious composites are less prone to moisture-related deterioration. Moreover, the consistency of the cementitious composite during preparation can influence the setting time and its performance. The consistency of cementitious composites can be adjusted for various applications to enhance efficiency and meet specific objectives.

**Figure 2:**
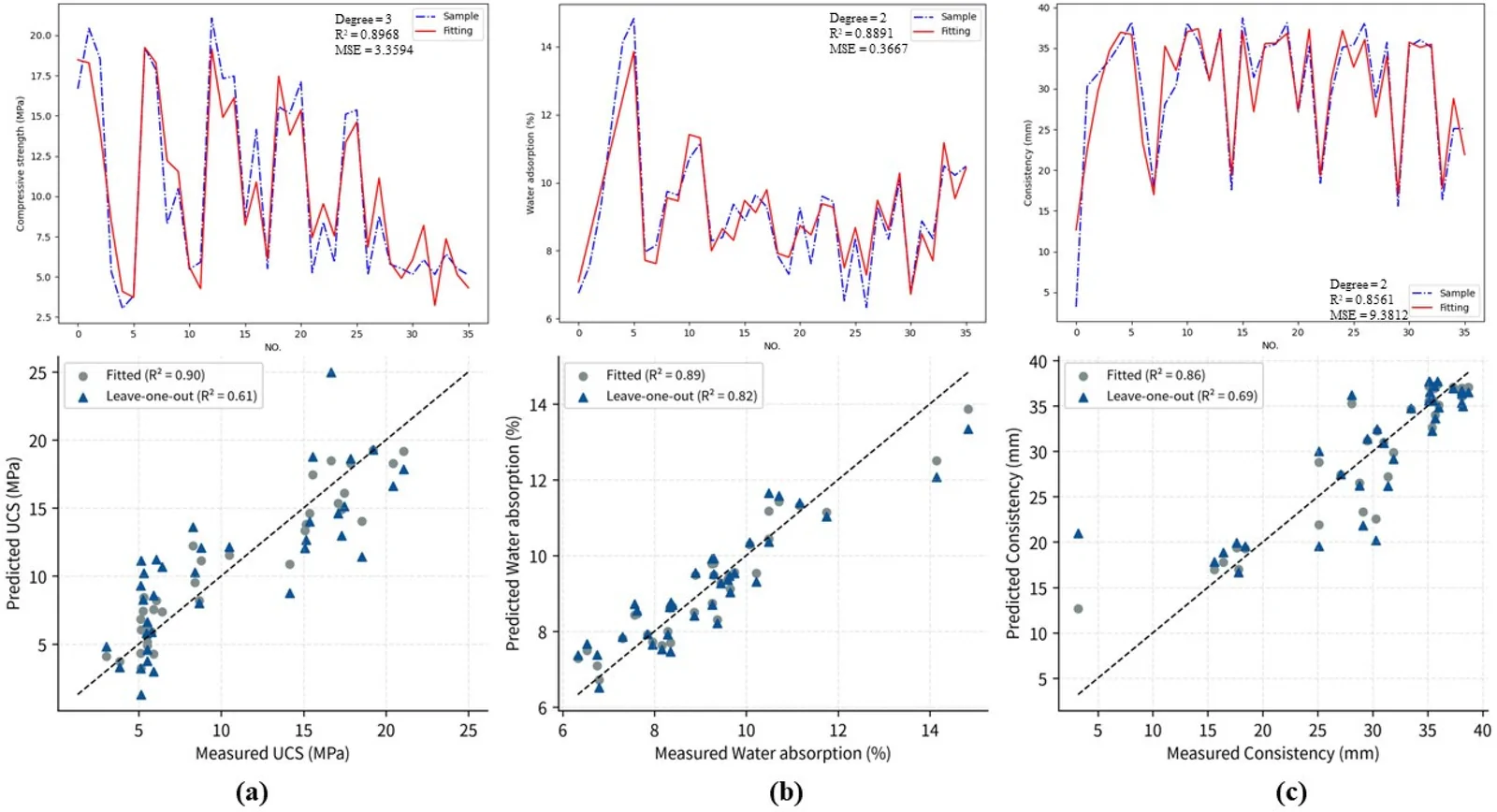
LASSO regression analysis for (**a**) UCS of polymer-modified cementitious composites; (**b**) water absorption, and (**c**) consistency.

The results of the orthogonal design were used as the training dataset to fit LASSO regression models in Python 3.12. The inputs were the three replacement percentages (FA-PFDS 0–50%, SYN 0–5%, CNF 0–2%), each standardized to zero mean and unit variance; the standardized inputs were expanded to polynomial terms (degree 3 for UCS, and degree 2 for water absorption and consistency) and then fitted with an *L*_1_ penalty (α = 0.1). The variables in Equations (3)–(5) are therefore standardized replacement percentages and not masses, and the coefficients are comparable across variables. The mean squared error (MSE) and R2 of the fits are given in Figure 2a–c: R^2^ = 0.897 for UCS, 0.889 for water absorption and 0.856 for consistency. Because these are in-sample values for a small dataset with many polynomial terms, a leave-one-out cross-validation (LOO-CV) was added. The LOO-CV R^2^ values were 0.62 (UCS), 0.82 (water absorption) and 0.69 (consistency), with the UCS model clearly less reliable than the in-sample statistics suggest. The models are therefore suitable for identifying trends and for screening candidate mixtures.

In Figure 2c, the relationship between the content of three different polymers and consistency of cement mortar is shown as the following model (Equation (3)), in which m_FA-PFDS_, m_SYN_ and m_CNF_ are standardized replacement percentages:

(3) $$\begin{array}{ll} \mathrm{Consistency}\;= & \;5.87\times m_{\mathrm{CNF}}-1.06\times m_{\mathrm{FA}-\mathrm{PFDS}}^{2}-1.23\times m_{\mathrm{FA}-\mathrm{PFDS}}\times m_{\mathrm{SYN}}-0.36\times m_{\mathrm{FA}-\mathrm{PFDS}}\times m_{\mathrm{CNF}}\\ & -0.55\times m_{\mathrm{SYN}}^{2}-0.13\times m_{\mathrm{SYN}}\times m_{\mathrm{CNF}}-3.01\times m_{\mathrm{CNF}}^{2}\\ & +34.86 \end{array}$$

The coefficient of 5.87 indicated that the proportion of CNF had a strong positive effect on consistency. The large positive coefficient suggested that as the proportion of CNF increased, the consistency of cementitious composites significantly increases. The high water absorption of CNF has also been reported in previous studies, in which a small proportion of CNF replacing cement significantly increased the consistency of cement mortar. However, previous studies [7,8] determined the superhydrophobicity of FA-PFDS and inert property of SYN. The interaction effects of adding proportional FA-PFDS and SYN suggested a more complex and negative impact on consistency when combined with each other. Therefore, consistency of cementitious composites was influenced by various factors including the type and proportion of polymers, water content, and the physical properties of components. Moreover, consistency affects the workability, water retention, and overall performance of the cementitious composites [25,26]. It determines that the cementitious composites can be mixed, applied, and shaped, making it a crucial property to control for achieving high-quality construction outcomes [27,28].

**Figure 3:**
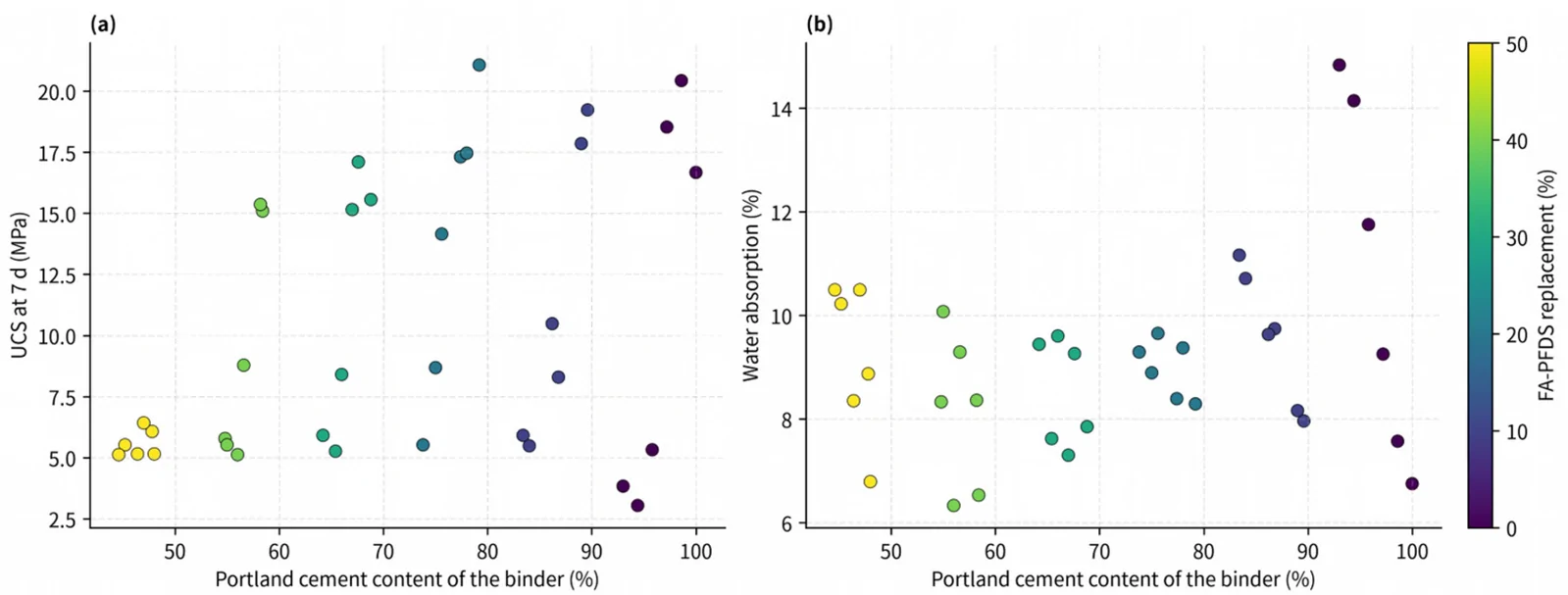
(**a**) 7-day UCS and (**b**) water absorption of the 36 mixtures versus the cement content of the binder (water-to-binder ratio 0.50 in all mixtures), colored by FA-PFDS replacement.

Effect of the cement content of the binder. The three modifiers replace cement mass for mass, so the water-to-binder ratio was 0.50 in all 36 mixtures (Table 1) and the replacement acts mainly by reducing the cement content of the binder from 100% to 44.6% (Figure 3). The cement content is almost perfectly collinear with the FA-PFDS replacement (r = −0.99) and is correlated with UCS (r = 0.40) and water absorption (r = 0.26), so these correlations cannot be separated from the effect of FA-PFDS (r with UCS = −0.32). A constant water-to-binder ratio does not make the mixtures equivalent in the water available to the reactive phase: fly ash reacts slowly at 7 days, FA-PFDS is hydrophobic and CNF binds water, so the water per unit mass of cement still rises from 0.50 to 1.12. The strength loss at high replacement is therefore partly a dilution effect of the reactive cement and cannot be attributed to the polymers alone.

In Figure 2b, the relationship between the content of three different polymers and water absorption of cementitious composites is shown as Equation (4):

(4) $$\begin{array}{ll} \mathrm{Water}\;\mathrm{absorption}\;=\; & -0.49\times m_{\mathrm{FA}-\mathrm{PFDS}}+0.48\times m_{\mathrm{SYN}}-0.002\times m_{\mathrm{CNF}}+0.48\times m_{\mathrm{FA}-\mathrm{PFDS}}^{2}\\ & -0.26\times m_{\mathrm{FA}-\mathrm{PFDS}}\times m_{\mathrm{SYN}}-0.99\times m_{\mathrm{FA}-\mathrm{PFDS}}\times m_{\mathrm{CNF}}+0.006\times m_{\mathrm{SYN}}\times m_{\mathrm{CNF}}\\ & +8.7 \end{array}$$

Moreover, the relationship between the content of the three polymers and UCS (Figure 2a) is shown as Equation (5):

(5) $$\begin{array}{ll} \mathrm{UCS}\;= & \;-0.7*m_{\mathrm{FA}-\mathrm{PFDS}}-4.92*m_{\mathrm{SYN}}-3.69*m_{\mathrm{CNF}}-1.38*m_{\mathrm{FA}-\mathrm{PFDS}}^{2}+0.54*m_{\mathrm{FA}-\mathrm{PFDS}}*m_{\mathrm{SYN}}+0.01\\ & *m_{\mathrm{FA}-\mathrm{PFDS}}*m_{\mathrm{CNF}}+0.42*m_{\mathrm{SYN}}^{2}-0.37*m_{\mathrm{SYN}}*m_{\mathrm{CNF}}-0.41*m_{\mathrm{FA}-\mathrm{PFDS}}^{3}+0.33\\ & *m_{\mathrm{FA}-\mathrm{PFDS}}^{2}*m_{\mathrm{SYN}}-0.51*m_{\mathrm{FA}-\mathrm{PFDS}}^{2}*m_{\mathrm{CNF}}+0.72*m_{\mathrm{FA}-\mathrm{PFDS}}*m_{\mathrm{SYN}}*m_{\mathrm{CNF}}-0.64\\ & *m_{\mathrm{FA}-\mathrm{PFDS}}*m_{\mathrm{CNF}}^{2}+0.45*m_{\mathrm{SYN}}^{3}+0.83*m_{\mathrm{SYN}}^{2}*m_{\mathrm{CNF}}+0.03*m_{\mathrm{SYN}}*m_{\mathrm{CNF}}^{2}+0.91*m_{\mathrm{CNF}}^{3}\\ & +11.87 \end{array}$$

The main effects of FA-PFDS (−0.49 × m_FA-PFDS_ and 0.48 × m^2^_FA-PFDS_) indicated that while the addition of FA-PFDS initially reduced water absorption, higher proportion of FA-PFDS increased it. A previous study [7] reported that superhydrophobic FA-PFDS can disperse well in cementitious composites and produce a denser matrix with fewer voids. This is a possible explanation of the reduced water absorption at low dosage. However, with the continuously increase of FA-PFDS, the cement content of the binder decreased and the Si/Al balance of the reaction products may have changed, which may have led to a more porous, lower-strength matrix. This structure can increase the water absorption. Moreover, the negative interaction effects (−0.99 × m_FA-PFDS_ × m_CNF_) suggested that FA-PFDS may reduce water absorption more effectively when combined with CNF.

The negative main effects of adding CNF and SYN (−4.92 × m_SYN_ and −3.69 × m_CNF_) indicated a reduction in UCS with increasing proportions of CNF and SYN. In order to ensure optimal UCS of a cementitious composite, the proportional amount of CNF and SYN should be controlled at a small content, due to their low density, poor dispersion and the reduced cement content of the binder.

In summary, the LASSO models suggest that the performance of the composites is governed by coupled, non-linear interactions among the polymers rather than by independent additive effects. FA-PFDS at moderate dosage was associated with lower water absorption while keeping the strength at an acceptable level. CNF and SYN may improve matrix compactness and interfacial behavior at low dosages, but excessive dosages reduced UCS. These interactions have to be considered for selecting mix proportions that balance strength and moisture resistance.

Given that UCS is a fundamental parameter for structural integrity, it was prioritized as the primary optimization target. All combinations of the tested levels were generated in Python and the UCS model was evaluated for each. The combination of 20% FA-PFDS, 1% SYN and 0.4% CNF was identified as the one that gave a high predicted UCS of 18.20 MPa with a cement reduction of 21.4%. The corresponding predictions of the other models were a water absorption of 8.15% and a consistency of 16.3 mm. This mixture is not one of the 36 tested mixtures, so these values are predictions and not measurements. They carry a prediction uncertainty at least as large as the LOO-CV error of the models. For comparison, the control reached 16.67 MPa with 6.75% water absorption, and the best measured mixture (20% FA-PFDS, 0% SYN, 0.8% CNF) reached 21.07 MPa with 8.29%. The predicted optimum therefore trades about 21% higher water absorption than the control for 21.4% less cement.

Among the combinations, 20% FA-PFDS, 1% SYN and 0.4% CNF has the highest predicted UCS among the combinations that contain all three polymers and at least 20% FA-PFDS. It is not the strength maximum of the model (20.5–21.6 MPa at 2% CNF without SYN), and the measured mixture (20% FA-PFDS, 0% SYN, 0.8% CNF) reached 21.07 MPa with a water absorption of 8.29% and a 20.8% cement reduction, which a higher strength than the predicted mixture at about the same absorption and cement saving. The predicted mixture was therefore chosen as a representative ternary composition and not because it is the best measured one. The leave-one-out root-mean-square errors were 3.5 MPa (UCS), 0.76% (water absorption) and 4.5 mm (consistency). A bootstrap of the whole fitting procedure gave 95% intervals for the predicted optimum of 13.4–19.5 MPa (UCS), 7.8–8.8% (water absorption) and 24.0–29.6 mm (consistency). These intervals reflect the dataset only and not the replicate scatter or model-form error. The regularization parameter was fixed at α = 0.1 and was not tuned. For α between 0.01 and 0.3, the leave-one-out R^2^ of the UCS model varied between 0.70 and 0.61 and the predicted UCS of the optimum between 18.4 and 16.3 MPa; for α = 1, the leave-one-out R^2^ fell to 0.43 (UCS) and 0.24 (water absorption).

### 3.2. Microstructural Observations and Proposed Mechanisms of the Polymer Combinations in Cementitious Composite

While the LASSO models quantified the effects of the polymers on strength and water absorption, XRD and SEM observations were used to discuss the physicochemical processes that may underlie these trends. The observations are qualitative and are interpreted as consistent with the proposed mechanisms.

**Figure 4:**
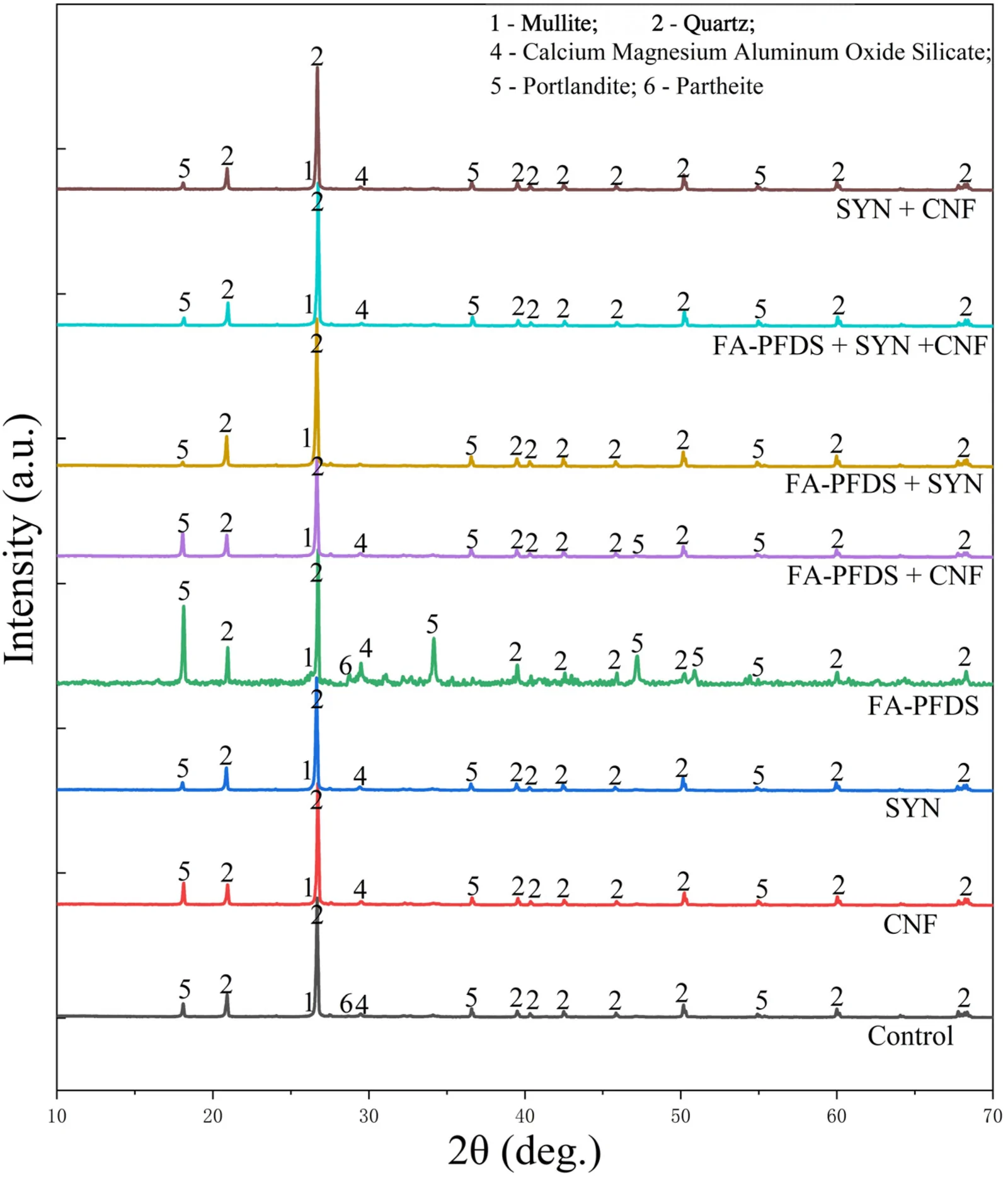
XRD patterns of cementitious composites with multiple polymers.

XRD patterns of cementitious composites with different compositions are shown in Figure 4. The control sample was a conventional cement specimen without modification of polymer. Peaks at 2θ values ranging from 10° to 70° were observed. The results of XRD analysis provide initial insight into the crystalline phase of cementitious composites.

The XRD analysis in previous study [7] determined that the major hydration products of cementitious composite included calcium silicate hydrates (C-S-H), calcium silicate aluminates hydrations (C-A-S-H) and composed of portlandite (C-H). With the addition of SYN and CNF, either individually or in combination, the diffraction patterns showed little change, which is consistent with these two polymers not forming new crystalline phases. XRD cannot detect the amorphous C-S-H gel or the polymer phases, so it does not exclude an effect on the hydration products. Previous studies suggested that CNF not only adjusted the consistency of cementitious composite but also functioned as a fine filler or linking agent similar to SYN, enhancing the cohesion of hydration products and reducing pore volume of cementitious composite. This may produce a denser matrix without changing the crystalline phases.

However, when proportional FA-PFDS was added alone, there was more intensity than in the control observed at 18°, 34°, 47°, and 51° (2θ) representing the portlandite peaks. This suggests that the mineral components of FA-PFDS changed the hydration products of the composite, but no pozzolanic reaction has been demonstrated. Portlandite peaks were weaker, but still present, in the patterns that contained CNF and SYN together with FA-PFDS. The decrease of the portlandite intensity could result from a lower cement content, a dilution effect, a different degree of hydration or the sample preparation. A quantitative analysis (Rietveld or TGA) is suggested to support any statement on portlandite consumption in future study.

**Figure 5:**
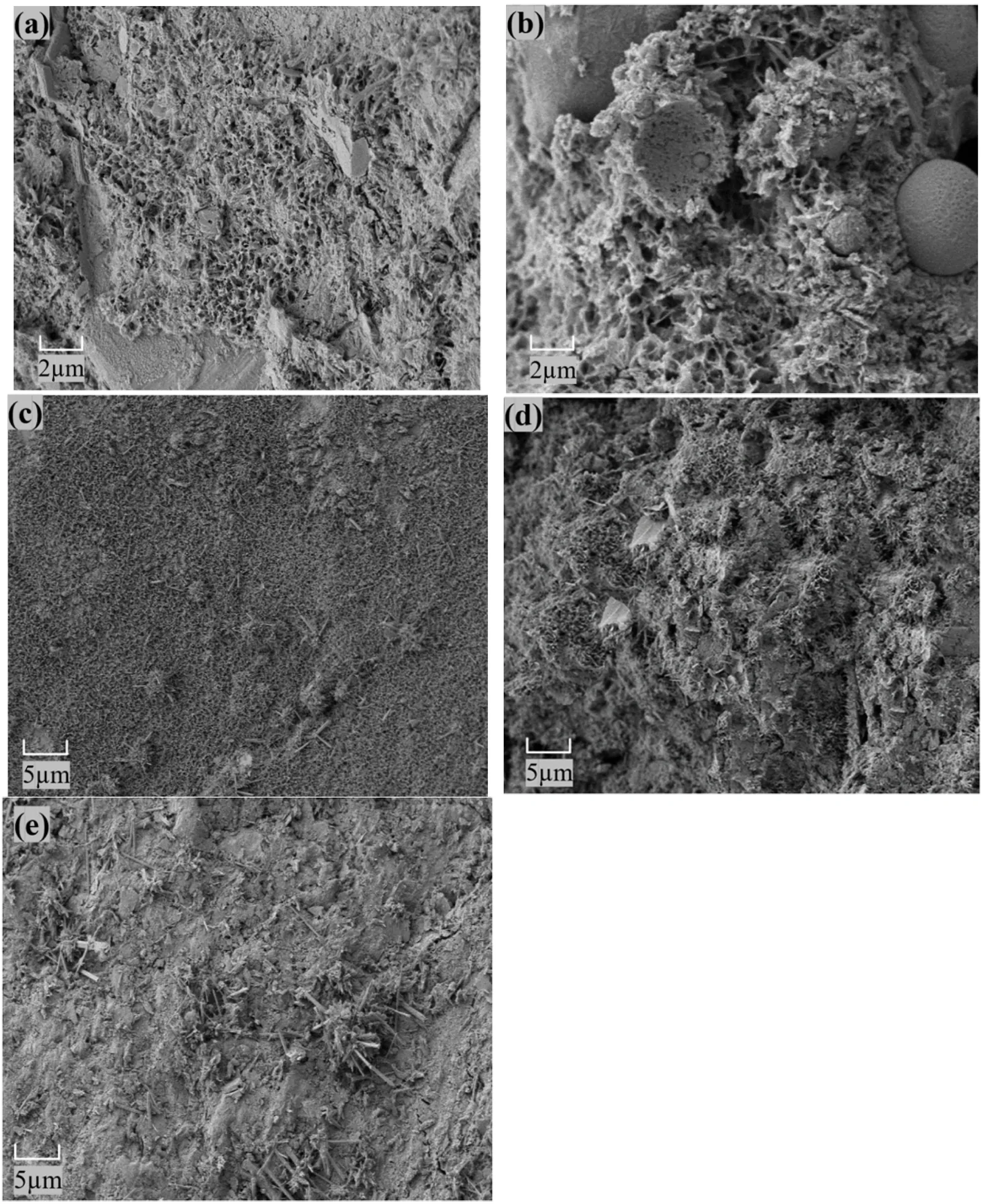
SEM images of (**a**) cement specimen (control); cementitious composite containing (**b**) FA-PFDS, (**c**) CNF, (**d**) SYN, and (**e**) multiple polymers.

A previous study [7] observed spherical FA-PFDS particles of various sizes (Figure 5b) that appeared to interact with the cementitious materials; the formation of C-A-S-H by pozzolanic reaction was proposed in that study. Moreover, the SEM images of CNF- and SYN-based cement specimens are shown in Figure 5c,d. The surface of SYN appeared coated with hydration products, which is compatible with a bridging interaction. Nano-scaled CNF may enter small pores and fill voids. In Figure 5e, the surface of FA-PFDS appeared coated with CNF and surrounded by SYN, and an increase in surface roughness can be seen, which could enlarge the contact area with the hydration products.

Although the combination of FA-PFDS, CNF, and SYN enhanced the surface roughness of FA-PFDS and increased its interaction with cementitious materials, excessive amounts of CNF and SYN may create a barrier layer that could be isolated FA-PFDS from the cementitious materials and limit its participation in hydration and pozzolanic reactions. It is therefore advisable to control the proportion of CNF and SYN used. Their main effect appears to be to regulate the water content of the cementitious composite, whereas FA-PFDS may contribute to the structural stability of the cementitious composite. Therefore, an optimal balance must be maintained to ensure that CNF and SYN improve the consistency and compactness of the cementitious composite without negatively affecting the functional contribution of FA-PFDS in the hydration process.

**Figure 6:**
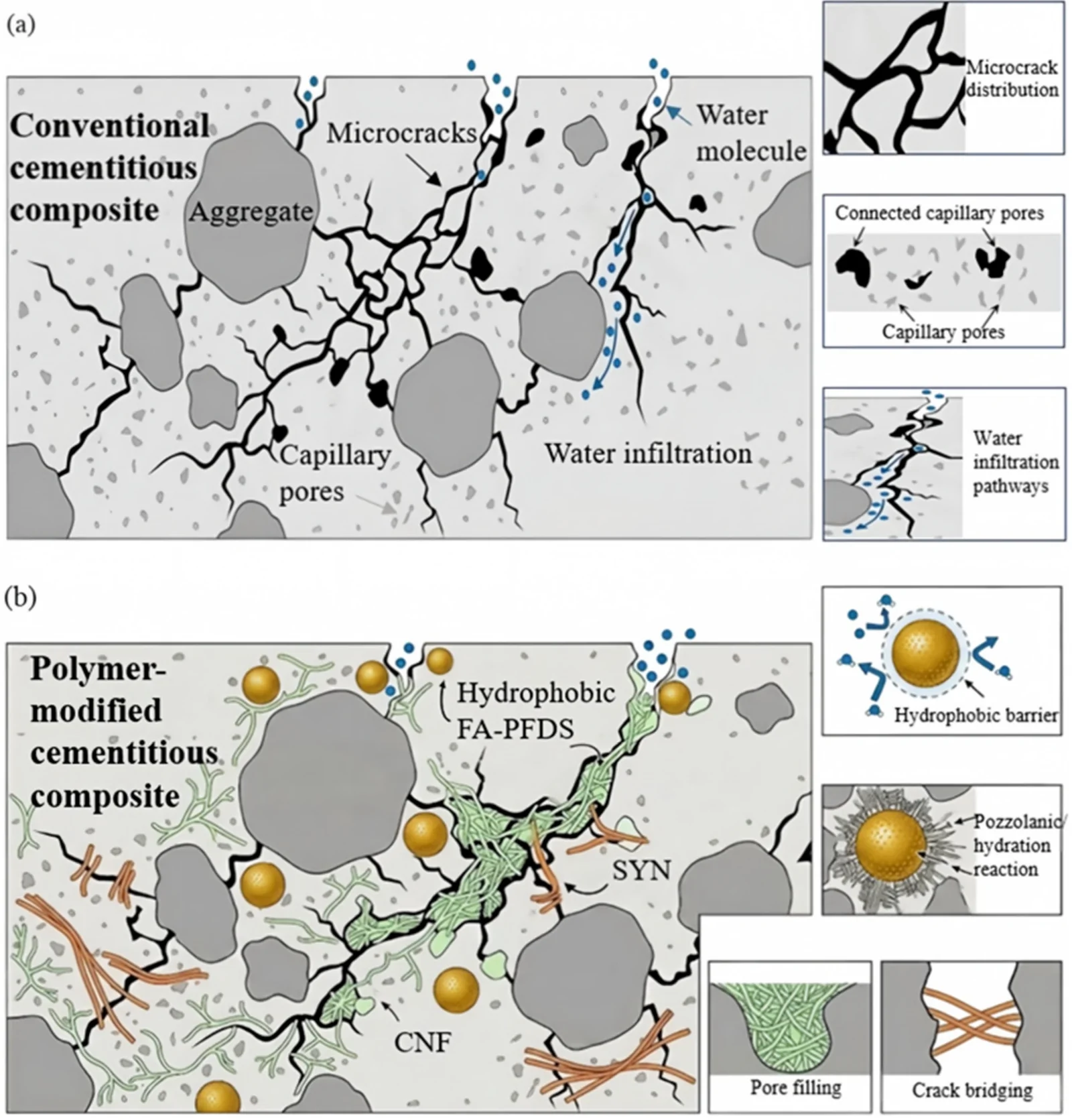
Proposed mechanism of FA-PFDS, CNF and SYN in cementitious composite.

The proposed mechanism appears to be dosage dependent. At suitable contents, CNF fills capillary pores, SYN improves interfacial crack bridging and FA-PFDS contributes hydrophobic and pozzolanic effects (Figure 6). Together they might form a denser matrix that improves moisture resistance while maintaining the mechanical performance. At excessive CNF or SYN contents, however, a barrier layer may isolate FA-PFDS from the cementitious matrix.

### 3.3. Comparative Assessment of Engineering Application Potential Using a Simplified Hydro-Mechanical Model

To explore the potential for engineering application, a simplified hydro-mechanical model was used to compare how a conventional concrete surface layer and the modified surface layer respond to 12 h of rainfall infiltration and to a 20 MPa static load. The modelling is a comparative sensitivity analysis and does not validate the structural reliability of the material.

**Figure 7:**
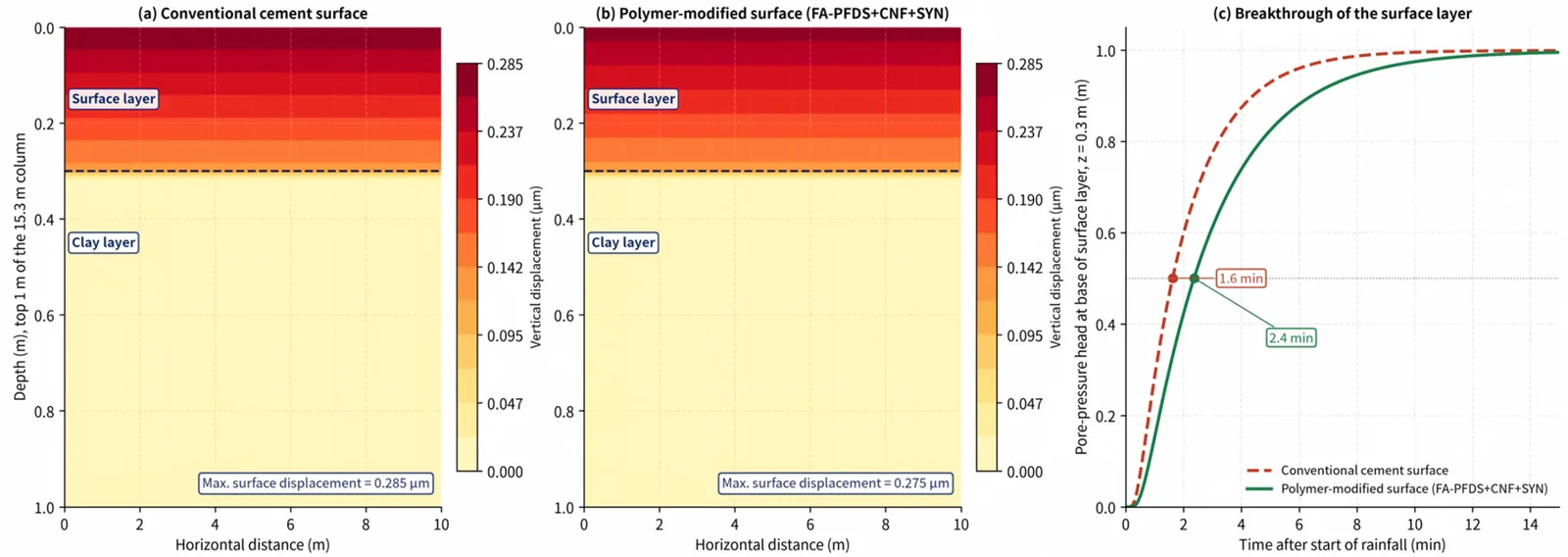
Vertical displacement (µm) caused by 12 h of rainfall infiltration for (**a**) the conventional concrete surface layer and (**b**) the polymer-modified surface layer, from the simplified one-dimensional hydro-mechanical model (common color scale, top 1 m shown). (**c**) Pore-pressure head at the base of the 0.3 m surface layer, normalized by the applied head, versus time; dots mark 50% breakthrough.

The simulation of continuous rainfall over a 12 h period compared the infiltration into the two slope structures. Water infiltrated each layer vertically under gravity. Displacements are expressed as the vertical displacement caused by the pore-pressure change. Figure 7a,b show the vertical displacement fields in the top 1 m of the conventional and modified columns with a common color scale, and Figure 7c shows the rise of the pore-pressure head at the base of the 0.3 m surface layer.

With a 0.3 m surface layer, both layers are saturated within minutes, so the wetting-front depth no longer separates them. The pore-pressure head at the base of the surface layer reached 50% of the applied head after 1.63 min for the conventional layer and 2.37 min for the modified layer (about 45% later), and 90% after 4.43 and 6.47 min, because the permeability of the modified layer was assumed to be 50% lower (Figure 7c). The maximum surface vertical displacement after 12 h was 0.275 µm for the modified layer and 0.285 µm for the conventional layer (Figure 7a,b). The delay depends strongly on the assumed permeability reduction: the 50% breakthrough time of the modified layer would be 1.2 min without a reduction, 2.37 min for 50% and 11.8 min for 90%.

**Figure 8:**
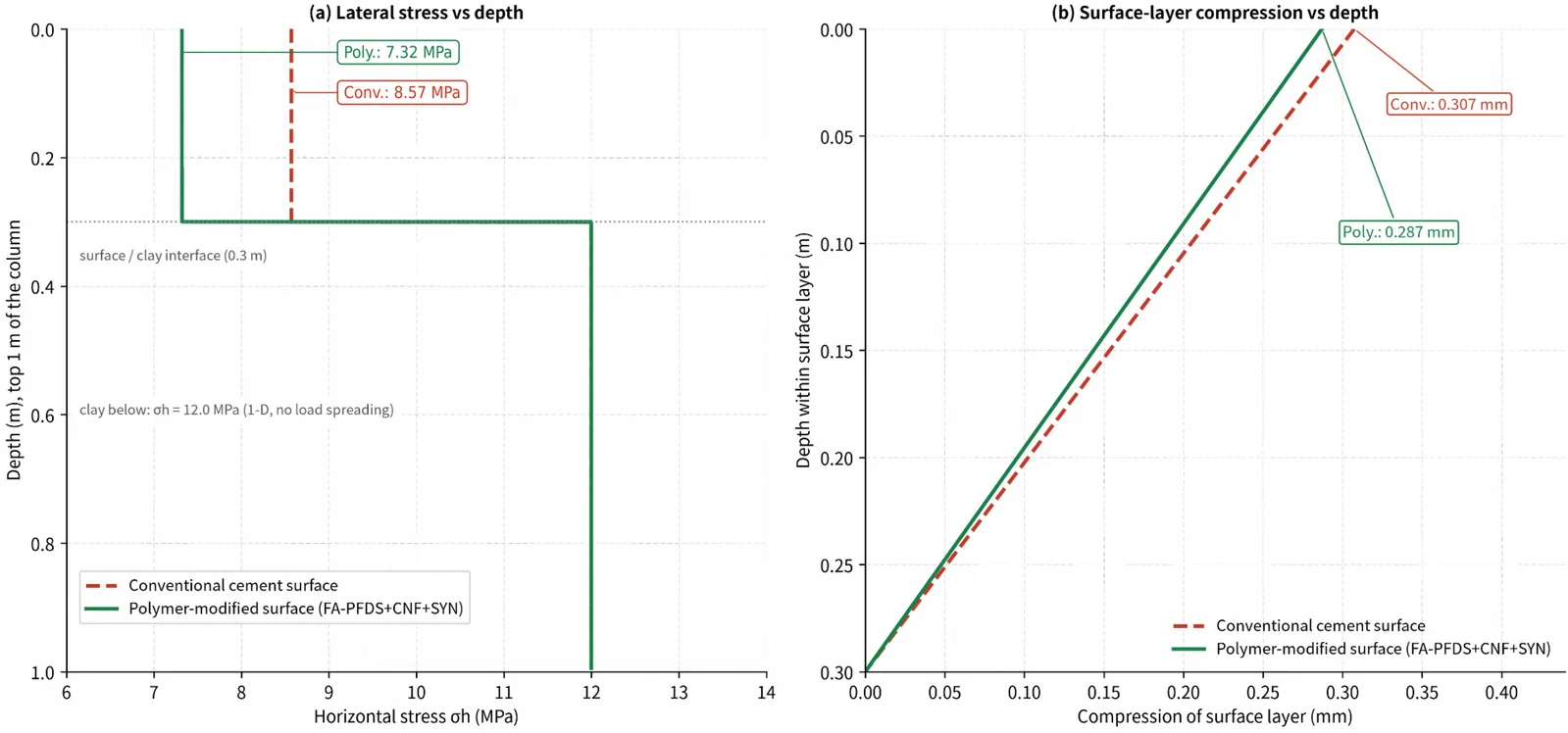
(**a**) Horizontal stress versus depth (top 1 m) and (**b**) elastic compression of the 0.3 m surface layer under a 20 MPa static load.

In the static loading analysis, a vertical stress of 20 MPa was applied to the surface layer. This level is of the order of the 7-day compressive strength of the mortar (16.7 MPa for the control and a predicted 18.2 MPa for the modified mixture), and is used as an extreme load case. The horizontal stress at the top of the surface layer under zero lateral strain was 8.57 MPa for the conventional layer and 7.32 MPa for the modified layer, and the elastic compression of the 0.3 m surface layer was 0.307 mm and 0.287 mm, respectively (Figure 8). The lower values of the modified layer follow from its higher assumed modulus (16,800 vs. 14,500 MPa) and lower Poisson’s ratio (0.268 vs. 0.30).

Overall, under the assumptions of the simplified model, the modified surface layer delays breakthrough of the thin surface layer and gives lower lateral stresses than the conventional layer for the same loads. The conclusion depends on the assumed material properties and must be confirmed with the full Abaqus model and with measured material data. The polymers derived from solid wastes can reduce the cement content, which may reduce environmental impacts.

## 4. Conclusions

This study investigated the proportions of FA-PFDS, CNF, and SYN as partial cement replacements in cementitious composites and their combined effects on mechanical properties and water absorption. A hybrid approach combining orthogonal mix design, LASSO regression, XRD/SEM observation and a simplified hydro-mechanical model was used. The main conclusions are as follows:

(1) LASSO regression described the non-linear interactions among the polymers within the tested range. FA-PFDS at moderate content was associated with lower water absorption, whereas CNF and SYN mainly affected the consistency and reduced the UCS at higher dosages. The model predicted a mixture of 20% FA-PFDS, 1% SYN and 0.4% CNF with a 21.4% reduction of cement, a 7-day UCS of 18.20 MPa and a water absorption of 8.15%. This is a model prediction, and the leave-one-out R2 of the models shows that the prediction is uncertain. The water absorption of the predicted mixture is higher than that of the control.
(2) XRD and SEM observations are consistent with pore filling by CNF and crack bridging by SYN and with an influence of FA-PFDS on the portlandite content. These observations are qualitative; the proposed mechanism, including a barrier layer around FA-PFDS at excessive CNF and SYN contents, is a hypothesis that needs quantitative verification.
(3) A simplified one-dimensional hydro-mechanical model, with corrected Poisson’s ratios and partly assumed properties, indicated that a surface layer with lower permeability delays water breakthrough of a 0.3 m surface layer (50% breakthrough after 2.4 vs. 1.6 min) and that the modified layer develops slightly lower lateral stress and compression under a 20 MPa load than the conventional layer. These results are comparative and depend on assumed parameters.

Several limitations should be considered. Firstly, the LASSO models were trained on 36 mixtures of the *L*_36_(6^3^) orthogonal design at a constant water-to-binder ratio of 0.50, so the cement content of the binder from 100% to 44.6% is confounded with the polymer dosage. Secondly, performance was assessed at an age of 7 days, after oven drying at 105 °C and 24 h of immersion; durability, including the alkaline stability of CNF and the SYN-matrix interface, freeze–thaw, wetting–drying and chemical attack remain to be investigated. Thirdly, the materials were not fully characterized, XRD was qualitative only and the mechanism is a hypothesis. Finally, environmental risks of the perfluorinated surface treatment must also be evaluated.

Overall, this study identifies a candidate low-cement mixture and discusses how it may balance UCS and water absorption within the tested range. With experimental confirmation, durability testing and field-scale validation, this approach may support the development of lower-carbon cementitious composites for engineering applications.

## Figures and Tables

**Table 1:** *L*_36_(6^3^) orthogonal design table used for preparing cementitious composites.

NO.	FA-PFDS (g)	SYN (g)	CNF (g)	Cement (g)	Sand (g)	Water (g)
1	0	0	0	603	1809	301.5
2	0	6.03	2.4	594.57	1809	301.5
3	0	12.06	4.8	586.14	1809	301.5
4	0	18.09	7.2	577.71	1809	301.5
5	0	24.12	9.6	569.28	1809	301.5
6	0	30.15	12	560.85	1809	301.5
7	60.3	0	2.4	540.3	1809	301.5
8	60.3	6.03	0	536.67	1809	301.5
9	60.3	12.06	7.2	523.44	1809	301.5
10	60.3	18.09	4.8	519.81	1809	301.5
11	60.3	24.12	12	506.58	1809	301.5
12	60.3	30.15	9.6	502.95	1809	301.5
13	120.6	0	4.8	477.6	1809	301.5
14	120.6	6.03	9.6	466.77	1809	301.5
15	120.6	12.06	0	470.34	1809	301.5
16	120.6	18.09	12	452.31	1809	301.5
17	120.6	24.12	2.4	455.88	1809	301.5
18	120.6	30.15	7.2	445.05	1809	301.5
19	180.9	0	7.2	414.9	1809	301.5
20	180.9	6.03	12	404.07	1809	301.5
21	180.9	12.06	2.4	407.64	1809	301.5
22	180.9	18.09	9.6	394.41	1809	301.5
23	180.9	24.12	0	397.98	1809	301.5
24	180.9	30.15	4.8	387.15	1809	301.5
25	241.2	0	9.6	352.2	1809	301.5
26	241.2	6.03	4.8	350.97	1809	301.5
27	241.2	12.06	12	337.74	1809	301.5
28	241.2	18.09	2.4	341.31	1809	301.5
29	241.2	24.12	7.2	330.48	1809	301.5
30	241.2	30.15	0	331.65	1809	301.5
31	301.5	0	12	289.5	1809	301.5
32	301.5	6.03	7.2	288.27	1809	301.5
33	301.5	12.06	9.6	279.84	1809	301.5
34	301.5	18.09	0	283.41	1809	301.5
35	301.5	24.12	4.8	272.58	1809	301.5
36	301.5	30.15	2.4	268.95	1809	301.5

Note: orthogonal design was carried out based on [14]. Masses of FA-PFDS, SYN and CNF replace the same mass of cement; replacement percentages refer to 603 g cement (binder = cement + FA-PFDS + SYN + CNF = 603 g in every mixture, so water/binder = 301.5/603 = 0.50).

**Table 2:** Numerical modeling parameters for different layers and polymers.

Structure Layer and Polymers	Density (kg/m^3^)	Modulus of Elasticity (MPa)	Poisson’s Ratio	Angle of Internal Friction (°)	Cohesion (kPa)	Porosity (%)	Permeability (cm/s)	Ref.
Surface layer (cement-based concrete)	2150–2400	13,000–16,000	0.25–0.35	41.7	29.8	19.1–43	0.43–1.8	[15,16,17]
Clay layer	1700–1991	500	0.35–0.40	20–30	27–75	31.97–47.3	<0.1	[15,18,19,20]
Foundation	1600–1800	50–60	0.35–0.45	25–30	56–150	32–37.6	<0.1	[17,19]
FA-PFDS	2200	23,000–30,000	0.16–0.21	38.5–41.9	42.8–69.7	20	<0.1	This study; [21,22]
CNF	1000–1500	3300–3500	0.38	-	-	-	-	This study; [23,24]
SYN	900–1500	5000–7000	0.35	-	-	-	-	This study; [23,24]
Modified surface layer (cement-based, 20% FA-PFDS, 1% SYN, 0.4% CNF)	2200	16,800	0.27	-	-	22.5	5.6 × 10^−3^	This study

**Table 3:** Corresponding results of orthogonal design for producing polymer-modified cementitious composites.

No.	UCS (MPa)	Water Absorption (%)	Consistency (mm)
1	16.67	6.75	3.2
2	20.43	7.57	30.3
3	18.53	9.25	31.9
4	5.32	11.75	33.5
5	3.04	14.14	35.6
6	3.83	14.83	38.2
7	19.23	7.96	29.1
8	17.85	8.16	17.8
9	8.29	9.74	28.1
10	10.48	9.63	30.4
11	5.48	10.71	38.1
12	5.91	11.16	35.9
13	21.07	8.29	31.0
14	17.31	8.39	37.3
15	17.46	9.37	17.6
16	8.68	8.89	38.7
17	14.15	9.65	31.4
18	5.52	9.29	35.1
19	15.56	7.85	35.5
20	15.15	7.30	38.1
21	17.10	9.26	27.1
22	5.26	7.62	35.2
23	8.40	9.60	18.4
24	5.91	9.44	29.5
25	15.09	6.53	35.1
26	15.36	8.36	35.4
27	5.12	6.33	38.1
28	8.78	9.29	28.8
29	5.78	8.33	35.7
30	5.52	10.07	15.6
31	5.15	6.79	35.2
32	6.07	8.87	36.0
33	5.15	8.35	35.1
34	6.42	10.49	16.4
35	5.52	10.22	25.1
36	5.12	10.49	25.1

## Data Availability

The original contributions presented in this study are included in the article. Further inquiries can be directed to the corresponding author.
